# Physiological and Transcriptomic Analyses of IAA-Induced Inhibition of Chlorophyll Formation in Potato Tubers Post-Harvest

**DOI:** 10.3390/foods14234031

**Published:** 2025-11-25

**Authors:** Hongze Lv, Fan Yang, Bidan Shi, Chuchu Zhang, Hui Ma, Jing Wang, Ke Shi, Guoqin Li, Yi Wu, Pengfei Zhang, Erihemu

**Affiliations:** Department of Food Science, Shanxi Normal University, Taiyuan 030006, China; lvhongze_sxun@126.com (H.L.); lemon58888@163.com (F.Y.); 18534857931@163.com (B.S.); chuchu20010909@126.com (C.Z.); huima@sxnu.edu.cn (H.M.); 15511816741@163.com (J.W.); 15735907356@163.com (K.S.); guoqin_li1@163.com (G.L.); wuyi1016@163.com (Y.W.); zhangpf@sxnu.edu.cn (P.Z.)

**Keywords:** potato greening, indole-3-acetic acid, chlorophyll content, storage, transcriptomics

## Abstract

Light exposure can induce post-harvest potato tubers to become green due to the accumulation of chlorophyll (Chl) and the formation of glycoside alkaloids, posing a potential risk to food safety. This study evaluated exogenous indole-3-acetic acid (IAA) to inhibit greening in *Solanum tuberosum* cv. Tongshu 31. IAA treatment maintained a relatively high a* value and significantly reduced both Chl content and the accumulation of toxic steroidal glycoalkaloids (SGAs), such as α-solanine and α-chaconine. In addition, IAA treatment also delayed the decrease in hardness and weight loss of tubers during storage, and helped maintain a high content of starch and reduce sugar. Transcriptome analysis revealed that IAA downregulated *HEMA1*, *CHLH*, and *GUN4*, among other key genes in the Chll biosynthesis pathway, thereby inhibiting Chl accumulation. IAA also modulated hormone networks, increasing gibberellin (GA) and jasmonic acid (JA), decreasing abscisic acid (ABA), and activating signaling genes (*StGID1*, *StJAR1*, *StPYR1*, *StPYL4*), enhancing tuber defense. These results indicate that IAA can inhibit the synthesis of Chl by regulating gene expression. This study provides a new strategy for alleviating the greening problem of potatoes and explains the potential mechanism by which IAA inhibits Chl synthesis in potato tubers.

## 1. Introduction

Potato (*Solanum tuberosum* L.) is the world’s fourth-largest staple crop after wheat, rice, and corn [1]. It provides essential nutrients such as starch, vitamins, and minerals [2]. However, exposure to light during supply chain processes induces tuber greening, a phenomenon involving chloroplast formation and chlorophyll (Chl) accumulation in subsurface cells [3,4,5]. This process stimulates the synthesis of toxic steroidal glycoalkaloids (SGAs) like solanine [6,7,8], which degrades tuber quality, raises food safety concerns, and causes significant economic losses [9]. Effective control of greening is thus essential to preserve both the nutritional and commercial value of potatoes.

In recent years, to address postharvest potato greening, researchers have developed a range of effective methods, including environmental factor control, chemical pre-treatment, and modified atmosphere packaging, all of which have demonstrated efficacy. For instance, Larsen and Molteberg [10] demonstrated that potato tubers stored at 5 °C do not exhibit greening after being subjected to light treatment for 14 days; conversely, those stored at 20 °C display significant Chl accumulation and subsequent greening. Dong et al. [11] reported that fumigation with 600 μL·L^−1^ ethanol (EF), along with EF combined with nitrogen (N_2_) treatment, can effectively reduce potato greening under illuminated conditions. Notably, EF-N_2_ treatment is more effective than EF alone. Air-conditioned storage has also emerged as an efficient method for minimizing photoinduced greening in potatoes. Banks [12] reported that treatment involving 5% O_2_ is more effective in inhibiting greening than treatments using either 15% CO_2_ or 21% O_2_. Furthermore, Nyankanga et al. [13] found that utilizing black perforated low-density polyethylene bags significantly prevents potato greening despite unavoidable exposure to light during storage and transportation. Chemical methods are important for controlling postharvest potato greening. Compounds such as herbicides, surfactants, and lecithin have been proven highly effective. However, they typically function by disrupting plastid structure and inhibiting Chl synthesis, which may adversely affect the tuber’s sensory qualities [14]. Thus, selecting chemical inhibitors that are environmentally friendly, easy to apply, and low-cost is crucial for effective greening control.

Exogenous phytohormone treatment has emerged as a promising natural postharvest strategy for maintaining the quality and extending the shelf life of fruits and vegetables during storage [15]. Recent studies have found that plant hormones such as melatonin (MT), salicylic acid (SA), gibberellin (GA), and abscisic acid (ABA) have shown good application potential in regulating Chl metabolism in fruits and vegetables. Qian et al. (2024) demonstrated that a 20 min treatment with 100 μM MT suppressed Chl biosynthesis in postharvest bamboo shoots [16]. Yu et al. (2024) found that spraying with 66.08 mg L^−1^ exogenous ABA significantly enhanced the expression of genes related to Chl degradation in loquat fruits, thereby inhibiting Chl accumulation [17]. However, Di et al. (2024) found that foliar spraying with 100 μM exogenous MT significantly delayed the yellowing of postharvest kale by repressing the expression of Chl-degrading enzyme genes [18]. Meanwhile, the research by Liu et al. (2025) demonstrated that foliar application of 2.5 mM exogenous SA effectively delayed Chl degradation in postharvest celery [19]. Furthermore, Keawmanee et al. (2022) pointed out that a single 500 μM GA foliar spray promoted the post-harvest “greenback” of Valencia orange fruits by upregulating Chl-synthesis genes and downregulating degradation-related genes [20].

Indole-3-acetic acid (IAA), as a ubiquitous plant hormone, plays a key role in the complex regulatory network of plant growth and development. The ability of IAA to regulate physiological processes ranging from cell division to fruit development makes it an effective strategy for extending the postharvest shelf life of various fruits and vegetables [21,22,23,24,25]. However, in terms of Chl metabolism regulation, the mechanism of action of IAA is complex and diverse. Ma et al. (2021) found that a 1 h treatment with 500 μM exogenous IAA significantly reduced the Chl content in citrus fruits [26], whereas Liu et al. (2025) found that foliar application of 2 mg L^−1^ exogenous IAA inhibited the expression of Chl-degradation-related genes in post-harvest Chinese cabbage [27]. Moreover, Wang et al. indicated that the accumulation of endogenous IAA could suppress Chl synthesis in potato tubers [6]. Therefore, the regulatory effect of exogenous IAA on Chl metabolism is dependent on the application concentration and duration.

While the functions of various phytohormones in Chl metabolism have been extensively studied, the transcriptional regulation of postharvest potato greening by exogenous IAA is poorly defined. Therefore, this study aims to determine the optimal IAA treatment concentration and duration for inhibiting the greening of potato tubers, and to systematically clarify its molecular mechanism through transcriptomics, thereby providing a theoretical basis for inhibiting the greening of post-harvest potatoes, which is of great significance for improving storage quality and industrial value.

## 2. Materials and Methods

### 2.1. Materials

Potatoes (*S. tuberosum* L. cv. Tongshu 31) were purchased from a local market in Taiyuan City and stored at 4 °C until use. Potatoes of similar sizes and free from damage were selected and randomly divided into two groups. The experiment was repeated three times. The potatoes were washed to remove surface deposits and air-dried naturally. IAA was purchased from Adamas-Beta Chemical Reagent Co., Ltd. (Shanghai, China), and all other reagents were of analytical grade.

### 2.2. Optimal Conditions for IAA Preprocessing

To determine the optimal IAA pretreatment conditions, we systematically evaluated soaking concentrations (0, 0.5, 1.0, 1.5, and 2.5 mM) and durations (0, 3, 6, 9, and 12 min) in a sequential manner. These levels were selected based on preliminary experiments. After the surface water was drained, the potatoes were placed in a light incubator (light intensity: 4000 lux, temperature: 25 ± 1 °C, and relative humidity RH: 75 ± 2%) and illuminated for 10 h to induce greening and evaluate the efficacy of IAA in inhibiting this process. Subsequently, the tubers were stored at room temperature (25 °C) away from light for 4 d. Samples were obtained on day 4 to assess the optimal soaking concentration and duration based on the a* value and Chl content of potato skins.

### 2.3. Experimental Treatment Method

**Figure 1:**
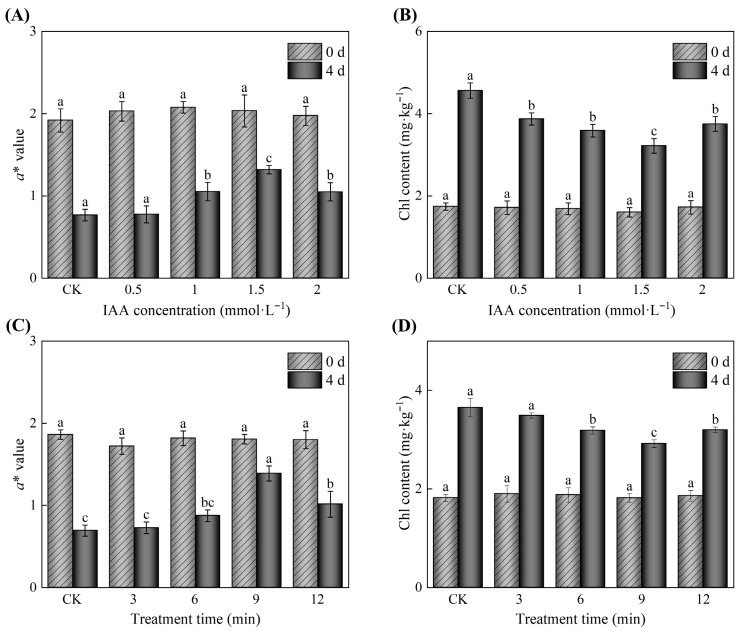
Effect of IAA treatment at varying concentrations and durations on the greenness of potato tubers stored at 25 °C for 4 days. (**A**) a* value of potato tubers treated with concentrations ranging from 0.5 to 2.0 mM; (**B**) chl content of potato tubers subjected to treatments with 0.5 to 2.0 mM; (**C**) a* value of potato tubers treated with 1.5 mM IAA for durations between 3 and 12 min; (**D**) chl content of potato tubers exposed to 1.5 mM IAA for periods ranging from 3 to 12 min. CK denotes the control group. Values are presented as standard error (*n* = 5) ± mean. The differences in treatment effects across each sampling time were statistically significant, with *p* < 0.05 indicated by different letters.

The optimal IAA treatment condition was determined to be 1.5 mM for 9 min based on the results of the single-factor experiments presented in Section 2.2, as this combination yielded the highest a* value and the lowest Chl content in potato peels after 4 days of storage (Figure 1), indicating the most effective inhibition of greening. Accordingly, a new batch of potato tubers was divided into three groups: (1) CKL (light control); (2) CKD (dark control); and (3) soak in 1.5 mM IAA for 9 min. Light induction was performed in the CKL and IAA soaking group according to the above methods. All groups were stored in the dark. Samples were obtained at days 0, 5, 10, 15, and 20 to assess greening and sensory quality parameters.

### 2.4. Evaluation of Potato Surface Color

The a* value of potato skin was determined following the method described by Dong et al. [11], with some modifications. The a* value indicates the degree of greening in the potato skin. In this study, 0.2 mm-thick peels were removed, after which the a* value was measured by using a CR-400 colorimeter (Guangdong Sanenshi Intelligent Technology Co., Ltd., Shenzhen, China).

### 2.5. Chl Extraction and Measurement

The Chl concentration in potato skin was measured according to the method of Sarkar et al. [28], with slight modifications. About 2 g of peel samples was combined with 6 mL of 95% ethanol and 0.1 g of calcium carbonate. The mixture was ground into a homogenate and extracted at 4 °C in the dark for 24 h. After centrifugation of the homogenate (10,000 rpm and 4 °C for 10 min), the supernatant was collected, to which additional 95% ethanol was added to reach a final volume of 10 mL, followed by thorough shaking. The absorbance of the supernatant was measured at 649 and 665 nm by using an ultraviolet–visible spectrophotometer (L5S, Shanghai Yidang Analytical Instruments Co., Ltd., Shanghai, China). The total Chl content was calculated using Formula (1).

(1) $$\mathrm{Chl}\;\mathrm{content}\;(\mathrm{mg}\cdot {kg}^{-1})=\frac{(18.08\;\times \;A_{649}+6.63\;\times \;A_{665})\;\times \;V}{W}$$

where A_649_ and A_665_ represent the absorption values of the supernatants at 649 and 665 nm, respectively; V is the volume of supernatant (mL); and W is the weight of cortex tissue (g). The total Chl content was estimated on a fresh weight basis.

### 2.6. Microscopic Observation

Microscopic observations were conducted following the methodology outlined by Zhang et al. [29], with slight modifications. Potato tubers were peeled to a thickness of 1.0 mm, and the underlying tissue was then manually sliced into sections as thin as possible (approximately 0.2 mm) using a scalpel. The slices were washed in distilled water, placed on a microscope slide, and covered with a cover glass for immediate examination under an inverted microscope (Leica Microsystems GmbH, Wetzlar, Germany). Images were captured in bright field at 100× and 400× magnification.

### 2.7. Determination of SGAs Content

The quantification of α-solanine and α-chaconine in potato tuber peel was performed according to a modified method derived from Zhang et al. (2025) [30]. Briefly, 1 g of peel sample was homogenized with 1 mL of deionized water by vortexing for 10 min, followed by extraction with 9 mL of acidified methanol (formic acid:methanol = 1:99, *v*/*v*) for 30 min. Thereafter, 0.25 g of anhydrous sodium sulfate and 0.3 g of anhydrous magnesium acetate were added. The mixture was vortexed for 10 min and then centrifuged at 8000× *g* for 10 min at 4 °C. A 100 μL aliquot of the supernatant was diluted to 10 mL with 40% (*v*/*v*) methanol–water and filtered through a 0.22 μm nylon membrane prior to analysis. Chromatographic separation was achieved using an Agilent Eclipse Plus C18 column (250 × 4.6 mm, 5 μm) maintained at 35 °C (Agilent, Santa Clara, CA, USA). The mobile phase consisted of (A) acetonitrile and (B) 0.01 M KH_2_PO_4_ buffer (pH 6.0), delivered at a flow rate of 1.2 mL/min. The injection volume was 10 μL. Detection was performed using mass spectrometry under positive electrospray ionization (ESI) mode (4000 V) with the ion source temperature set to 350 °C; simultaneous UV detection was carried out at 208 nm. Commercially available standards of α-solanine and α-chaconine (purity ≥ 98%, Shanghai Yuanye Biotechnology Co., Ltd., Shanghai, China) were used for identification and quantification based on retention time alignment and external calibration with peak areas.

### 2.8. Quality Parameters

#### 2.8.1. Fruit Firmness Test

The hardness was determined using the method of Emragi et al. [31], with some modifications. In short, hardness was measured using a texture analyzer (model TA.XT plus). The analyzer was equipped with a P/2 probe (diameter of 2 mm) with the weight (N) required to make a 10 mm indentation on the surface of the tuber. Each treatment group comprised three potatoes, and each potato was measured five times to calculate the average.

#### 2.8.2. Measurement of Weight Loss

The weight loss was calculated by Equation (2).

(2) $$Weight\;loss=\frac{\left(M_{0}-M\right)}{M_{0}}\times 100\%$$

where M_0_ is the fruit weight just before storage (g), and M represents the fruit weight after the storage period (g).

#### 2.8.3. Starch Determination

Approximately 2 g of potato pulp was weighed and 10 mL of 1% HCl was added. After grinding, the samples were hydrolyzed in a water bath for 60 min. Following neutralization with NaOH, 5 mL each of 4% zinc acetate and potassium ferricyanide were added. The samples were then filtered and stored. Approximately 2 mL of the sample was measured and 1.5 mL of DNS reagent was incorporated. The samples were heated, cooled, and diluted to a final volume of 10 mL. Absorbance was measured at 540 nm. The glucose content (*m*) was calculated using the calibration curve equation y = 0.51526x − 0.017. Calculation formula: The starch content was calculated according to Equation (3).

(3) $$Starch\;content\left(\%\right)=\frac{V\times m\times 0.9}{V_{s}\times M\times 1000}\times 100$$

where V is the total volume of sample extract (mL); m is the amount of glucose (mg) obtained by the standard curve; V_s_ is the volume of extracted liquid (mL) that reacts with DNS; and M is the sample mass (g).

#### 2.8.4. Determination of Reducing Sugar Content

For this section, the method of Nourian et al. [32] was referred to, with some modifications. Potato (2 g) was ground with 20 mL of water and the mixture was heated in a water bath at 80 °C for 30 min. The volume was adjusted to 25 mL, after which the mixture was cooled, filtered, and centrifuged at 1000 rpm and 4 °C for 10 min. The supernatant was collected and diluted fivefold. Two milliliters of the diluted sample was mixed with 1.5 mL of DNS reagent. The solution was boiled for 5 min, cooled, and then diluted to a final volume of 10 mL. Absorbance was measured at 540 nm. Glucose content (*m*) was calculated using the calibration curve equation y = 0.51526x − 0.017. The reducing sugar content was calculated by Equation (4).

(4) $$Reducing\;sugar\;content\left(\%\right)=\frac{V\times m}{V_{s}\times M\times 1000}\times 100$$

where V is the total volume of sample extract (mL); m is the amount of glucose (mg) obtained by the standard curve; V_s_ is the volume of extracted liquid (mL) reacting with DNS; and M is sample mass (g).

#### 2.8.5. Determination of Dry Matter Content

The dry matter content of potato pulp was measured according to the method of Sidauruk et al. [33], with some modifications. The peeled potatoes were cut into evenly sized and thick slices, each weighing about 2.5 g. Each potato slice was placed on an aluminum plate and dried in an oven at 80 ± 1 °C for 3 h. The initial and final weights of each potato slice were recorded. The dry matter content was calculated by Equation (5).

(5) $$Dry\;matter\;content=\frac{Dry\;Weight}{Tatol\;Weight}\times 100\%$$

### 2.9. RNA Extraction, Library Preparation, Sequencing, and Data Analysis

Transcriptomic analysis was performed on potato tuber flesh samples subjected to four treatments: CKL-0d, IAA-0d, CKL-20d, and IAA-20d. To preserve RNA integrity, all samples were flash-frozen in liquid nitrogen and ground to a fine powder prior to total RNA extraction using TRIzol reagent. Specifically, 100 mg of tissue powder was homogenized in 1 mL of TRIzol, followed by phase separation with 200 μL of chloroform and centrifugation at 12,000× *g* for 15 min at 4 °C. The aqueous phase was collected, and RNA was precipitated with an equal volume of isopropanol. The resulting RNA pellet was washed twice with 75% ethanol, air-dried, and resuspended in 30 μL of RNase-free water. RNA purity was assessed using a Nanodrop ND-2000, with samples required to have A260/A280 ratios between 1.9 and 2.1 and A260/A230 ratios ≥ 2.0. RNA integrity was evaluated using an Agilent 4150 Bioanalyzer, and only samples with an RNA Integrity Number (RIN) ≥ 7.0 were used for library construction.

Sequencing libraries were prepared using the ABclonal mRNA-seq Lib Prep Kit. Poly(A)+ mRNA was enriched from 1 μg of total RNA using oligo(dT) magnetic beads and fragmented using divalent cations at elevated temperature. First-strand cDNA was synthesized using random hexamer primers and M-MuLV Reverse Transcriptase (RNase H), followed by second-strand synthesis with DNA Polymerase I and RNase H. The double-stranded cDNA was subjected to end repair, adenylation at the 3′ ends, and ligation to Illumina sequencing adapters. Fragments of approximately 300 bp were selected and amplified by 15 cycles of PCR to construct the final cDNA libraries. Library quality and size distribution were assessed using an Agilent Bioanalyzer 4150 system. Qualified libraries were sequenced on an Illumina NovaSeq 6000 platform to generate 150 bp paired-end reads.

Raw sequencing data were processed with Fastp (v0.23.2) to remove adapter sequences and low-quality reads (those with >5% N bases or >60% of bases having a Phred score ≤25). Clean reads were aligned to the potato reference genome (https://dec2021-plants.ensembl.org/Solanum_tuberosum/Info/Index, 30 November 2024) using HISAT2 (v2.2.1). Gene expression levels were quantified as FPKM (Fragments Per Kilobase of transcript per Million mapped reads) using FeatureCounts (v2.0.3). Differential expression analysis was performed with DESeq2 (v1.34.0), applying thresholds of |log_2_(fold change)| > 1 and adjusted *p*-value < 0.05. Functional annotation was carried out using Blast2GO (v2.5.0) for Gene Ontology (GO) terms and kobas-annotate (v3.0.3) for KEGG pathways. Enrichment analysis was performed using GO.db (v3.14.0) and topGO (v2.46.0), with a significance threshold of *p* < 0.05.

### 2.10. Real-Time Quantitative PCR (RT-qPCR) Analysis

Six differentially expressed genes involved in Chl synthesis were validated by real-time quantitative PCR (RT-qPCR). Total RNA was extracted from frozen potato peels using the Plant RNA Kit (Omega Bio-Tek, Norcross, GA, USA), and its quality was confirmed (260/280 = 1.9–2.1, RIN ≥ 7.0). First-strand cDNA was synthesized from 1 µg RNA with the Genstar reverse transcription kit (GenStar Biosolutions, Beijing, China). RT-qPCR was performed on a CFX Connect™ system (Thermo Scientific, Waltham, MA, USA) in 20 µL reactions containing 2× SYBR Green Master Mix, 0.4 µM primers, and 1 µL ten-fold-diluted cDNA, yielding a final cDNA concentration of 0.25 ng/µL; cycling conditions were 95 °C for 30 s followed by 40 cycles of 95 °C for 5 s and 60 °C for 30 s. Each gene was analyzed in three biological replicates. StEF-1α served as the reference gene [34], and relative expression was calculated using the 2^−ΔΔCt^ method [23,35]. Primer sequences are listed in Table S1.

### 2.11. Quantification of Phytohormones (ABA, JA, and GA)

Phytohormones (ABA, JA, GA) analysis was performed by Metware Biotechnology Co., Ltd. (Shanxi, China) using an AB Sciex QTRAP 6500 LC-MS/MS platform according to a previously described methodology [36]. The experiment included four treatment groups: CK 0 d, IAA 0 d, CK 20 d, and IAA 20 d, each with three biological replicates. Briefly, 50 mg of freeze-ground potato powder was thoroughly extracted with 1 mL of methanol/water/formic acid (15:4:1, *v*/*v*/*v*). After centrifugation at 4 °C, the supernatant was collected, concentrated, and reconstituted in 100 μL of 80% methanol. The solution was filtered prior to UPLC-MS/MS analysis. Chromatographic separation was carried out on a Waters ACQUITY UPLC HSS T3 C18 column (1.8 μm, 2.1 mm × 100 mm) maintained at 40 °C. The mobile phase consisted of solvent A (ultrapure water with 0.04% acetic acid) and solvent B (acetonitrile with 0.04% acetic acid). The gradient elution program was set as follows: 95% A/5% B at 0 min, held until 1.0 min; a linear gradient to 5% A/95% B at 8.0 min, maintained until 9.0 min; returned to 95% A/5% B at 9.1 min and held until 12.0 min. The flow rate was 0.35 mL/min and the injection volume was 2 μL. Mass spectrometric detection was conducted using an electrospray ionization (ESI) source operated at 500 °C. The ion spray voltages were set at 5500 V (positive mode) and −4500 V (negative mode), with a curtain gas pressure of 35 psi. Phytohormones (ABA, JA, GA) were analyzed using scheduled MRM. Identification and quantification were performed using Metware’s in-house phytohormones database, which was established using authentic standards.

### 2.12. Statistical Analysis

All data were first subjected to one-way analysis of variance (ANOVA) using IBM SPSS v. 26.0 (SPSS Inc., Chicago, IL, USA). When the ANOVA indicated a significant difference (*p* < 0.05), Duncan’s multiple range test was applied to separate the means. All experiments were performed with five independent replicates, and the results are expressed as mean ± standard deviation.

## 3. Results and Discussion

### 3.1. Determination of the Optimal Conditions for IAA Treatment

The a* value represents the magnitude of color change between red (+) and green (−), and the magnitude of a* can reflect greening in potato tubers. The effect of IAA treatment concentrations on the a* value of potato tubers is shown in Figure 1A. The results indicated that the a* values of all treatment groups significantly decreased on day 4 with the extension of storage time (*p* < 0.05). However, the a* value of potato tubers treated with IAA was significantly higher than that of the CK groups (*p* < 0.05). Moreover, a* of the 1.5 mM IAA treatment group was significantly higher than that of the other treatment groups (*p* < 0.05), representing a 72.52% increase compared with the CK group after 4 days of storage. As shown in Figure 1B, the Chl content of potato tubers treated with IAA initially decreased and then increased with rising IAA concentration, and it was significantly higher than that of the CK group (*p* < 0.05). This result was consistent with the changes in a* value of potato tubers (Figure 1A). The lowest Chl content of IAA-treated potato tubers was 3.22 mg·kg^−1^ at 1.5 mM IAA, indicating a decrease of 29.52% as compared with the CK groups, respectively, after 4 days of storage.

The effect of IAA treatment time on the a* value of potato tubers is shown in Figure 1C. The potato tubers treated with IAA at different durations were able to maintain their a* values. However, as the treatment time increased, the a* values initially rose and then declined. Additionally, when the IAA treatment duration was 9 min, the a* value of potato tubers reached the highest level compared with the other treatment groups (*p* < 0.05). The changes in the Chl content of potato tubers under different IAA treatment times are shown in Figure 1D. The Chl content of potato tubers treated with IAA for 9 min was significantly lower than that of other treatments (*p* < 0.05). In particular, the Chl content of potato tubers treated with IAA for 9 min remained 20% lower than that of the CK group. These results showed that the optimal conditions for inhibiting greening in potato tubers using IAA were a concentration of 1.5 mM and a treatment duration of 9 min.

**Figure 2:**
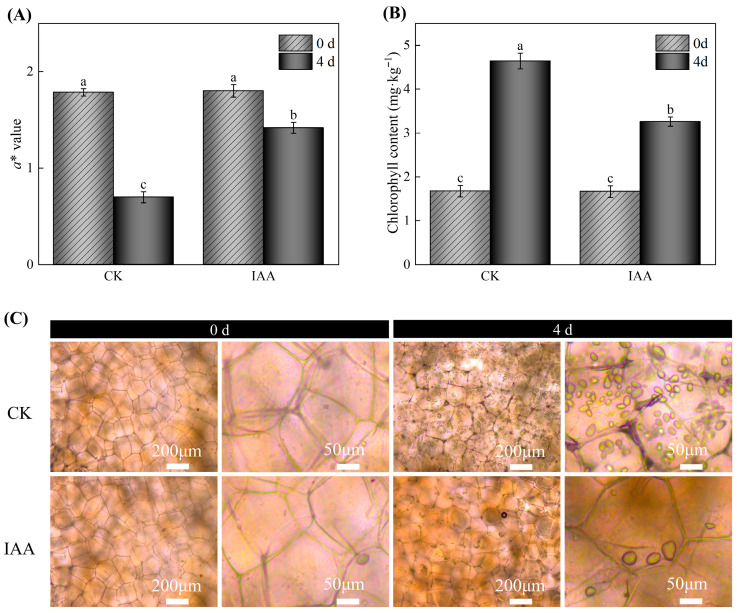
Effects of IAA treatment on the a* value (**A**), chl content (**B**), and microscope images (**C**) of potato tubers on the fourth day of storage are shown. CK represents the control group. The values are presented as the mean ± standard error (*n* = 5). Different letters indicate significant difference in the treatment of each sampling time at *p* < 0.05.

To ensure the reliability and scientific validity of the experimental results, this study conducted a verification experiment based on the optimal IAA treatment protocol determined from single-factor experimental results. As shown in Figure 2A, a notable decline in the a* values of potato tubers was observed on day 4, indicating the progressive development of greening. Notably, the IAA-treated group maintained significantly higher a* values than the control group at day 4 (*p* < 0.05), highlighting the efficacy of IAA in mitigating greening. In Figure 2B, the Chl content increased during storage, but IAA-treated potatoes exhibited a 29.73% reduction in Chl accumulation relative to the control samples. Microscopic examination revealed that after 4 days of storage, a faint greenish hue was barely discernible around the cell walls in IAA-treated samples, whereas distinct Chl deposition was evident in the control group (Figure 2C). This result was consistent with the changes in a* value and Chl content of potato tubers (Figure 2A,B). These findings collectively demonstrate the role of IAA treatment in reducing Chl accumulation and greening in potato tubers. Thus, an IAA concentration of 1.5 mM with a treatment duration of 9 min was selected for subsequent experimental investigations.

Extensive scientific investigations have established IAA as a crucial phytohormone that significantly influences various physiological and biochemical pathways in postharvest fruits and vegetables [15]. However, the regulatory effects of IAA treatment on the postharvest quality of fruits and vegetables exhibit significant concentration- and time-dependence. Zhou et al. [23] reported that treating mango fruits with 1.0 mM IAA for 5 min can enhance antioxidant capacity, delay postharvest senescence, and ultimately maintain high fruit quality. Similarly, Zhou et al. [22] demonstrated that 15 min of immersion in 0.5 mM IAA significantly enhances the cold tolerance of peaches while effectively maintaining their overall fruit quality. However, a recent study by Guan et al. [37] demonstrated that 15 min of immersion in 0.5 mM IAA solution significantly accelerates softening in kiwifruit, in contrast to previous findings on IAA’s role in delaying storage. Zhu et al. [38] also found that treating postharvest ‘Docteur Jules Guyot’ pears with 1.0 mM IAA for 30 min accelerates their ripening and softening. Additionally, the findings of this study indicated that the most effective inhibition of greening in potato tubers was obtained through treatment with 1.5 mM IAA for 9 min (Figure 1).

### 3.2. Effects of Different Treatments on the Overall Appearance, a* Value, and Epidermal Chl Content of Potato Tubers During Storage

**Figure 3:**
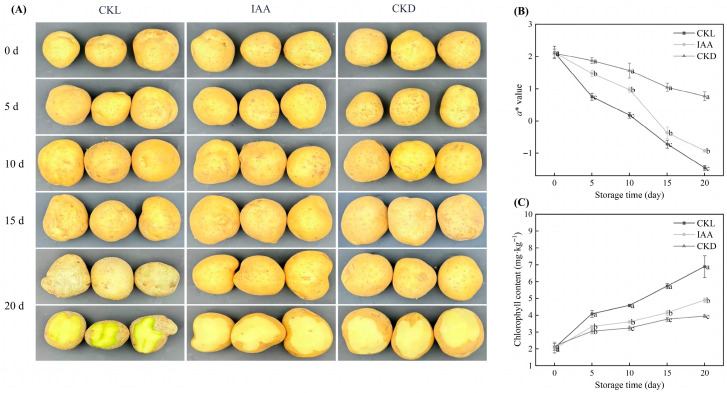
The figure illustrates the impact of IAA treatment on the appearance (**A**), a* value (**B**), and chl content (**C**) of potato tubers stored for 20 days. CKL represents the light control group, while CKD denotes the dark control group. Data are presented as mean ± standard error (*n* = 5). Different letters indicate significant difference in the treatment of each sampling time at *p* < 0.05.

The overall appearance changes in whole potato tubers subjected to different treatments during storage are shown in Figure 3A. At the initial storage stage (0 days), no significant differences were observed in the overall appearance of potato tubers. However, at 20 days of storage, IAA treatment demonstrated significantly better overall appearance compared with CKL-treated tubers. Meanwhile, the a* value of potato tubers decreased with storage time (Figure 3B). At 20 days of storage, the IAA-treated potato tubers maintained significantly higher a* values compared with the CKL samples (*p* < 0.05). The effect of IAA treatment on the Chl contents of whole potato tubers during storage is shown in Figure 3C. The Chl content of potato tubers increased with the extension of storage time. After 20 days of storage, compared with the initial storage period (0 days), the CKL, CKD, and IAA treatments exhibited significant increases in epidermal Chl content by 236.80%, 82.95%, and 144.65%, respectively. At the 20-day storage point, the Chl content of IAA-treated potato tubers was significantly lower than that of the CKL group (*p* < 0.05). In particular, the IAA-treated potato tubers still had a 29.04% lower Chl content than the CKL group at the 20-day storage point.

The greening of potato tubers is primarily attributed to the light-induced conversion of amyloplasts to chloroplasts in the periderm [39,40]. Research has demonstrated that light exposure upregulates the expression of key genes involved in Chl biosynthesis, including *HEMA1* (encoding glutamyl-tRNA reductase, the rate-limiting enzyme), *GSA*, *CHLH*, and *GUN4* [9]. Light promotes the transformation of amyloplasts to chloroplasts and activates the expression of Chl biosynthesis-related genes, ultimately leading to significant increases in Chl content. Exogenous IAA treatment significantly inhibits Chl synthesis by downregulating the expression of key Chl biosynthesis genes. Interestingly, the CKD-treated potato tubers exhibited better performance compared with the samples subjected to other treatments, indicating that light exposure is the predominant factor contributing to the greening of potato tubers. Consistent with the findings of Zhang et al. [29], light exposure not only promotes Chl synthesis in potatoes but also leads to an increase in Chl content with prolonged illumination. However, light exposure is inevitable during storage and transportation. Therefore, IAA treatment of potato tubers emerges as an effective method to mitigate greening.

### 3.3. Effects of IAA Treatment on the SGAs Content of Whole Potato Tubers During Dark Storage at Different Temperatures

**Figure 4:**
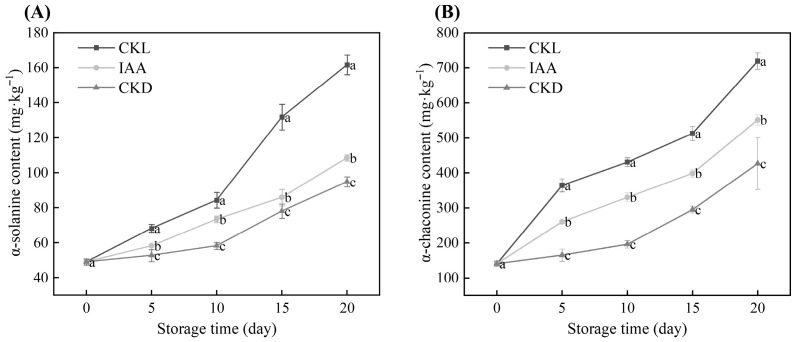
Effects of IAA treatment on α-solanine (**A**) and α-chaconine (**B**) content of potato tubers during different temperatures of storage. CKL represents the light control group, while CKD denotes the dark control group. Data are presented as mean ± standard error (*n* = 5). Different letters indicate significant difference in the treatment of each sampling time at *p* < 0.05.

As mentioned earlier, the photoinduced greenness of potato tubers is usually accompanied by the synthesis and accumulation of SGAs, among which α-solanine and α-chaconine account for more than 95% of the total content [41]. The changes in the contents of α-solanine and α-chaconine in potato tubers during storage under exogenous IAA treatment are shown in Figure 4. The contents of α-solanine and α-chaconine in tubers increased with the extension of storage time (Figure 4A,B). At 20 days of storage, compared with the initial level (0 days), the α-solanine content in the CKL group, IAA treatment group and CKD group increased by 229.18%, 120.89% and 93.19%, respectively (Figure 4A). The α-solanine content in tubers treated with IAA was significantly lower than that treated with CKL (*p* < 0.05), decreased by 32.89%, but was significantly higher than that in the CKD group (*p* < 0.05). Similarly, the α-chaconine content in tubers treated with IAA was significantly lower than that in the CKL group (*p* < 0.05), but still significantly higher than that in the CKD group (*p* < 0.05) (Figure 4B). This indicates that IAA treatment significantly inhibited the accumulation of SGAs in potato tubers. Rymuza et al. (2020) [42] and others’ previous studies have shown that light exposure can upregulate the expression of key biosynthase genes of SGAs, such as HMGR, SQS, and SGT1, leading to elevated levels of α-solanine and α-chaconine [42]. This study found that the SGAs content in the CKL group increased rapidly during the storage period and was significantly higher than that in the CKD group samples. This further confirms that light is one of the main environmental drivers for the accumulation of SGAs in tubers. In addition, the SGAs content in the IAA treatment group was significantly lower than that in the CKL group, indicating that IAA can regulate the biosynthesis or degradation process of SGAs, but its specific mechanism of action remains unclear. Subsequent studies will integrate transcriptomics and metabolomics to systematically clarify the regulatory mechanism of exogenous IAA on SGAs in potato tubers. Interestingly, even under dark storage conditions at 25 °C, the concentrations of α-solanine and α-chaconine in the CKD group increased, which was consistent with the research results of Dong et al. (2017) [11].

### 3.4. Exogenous IAA-Induced Quality Changes in Potato Tubers

**Figure 5:**
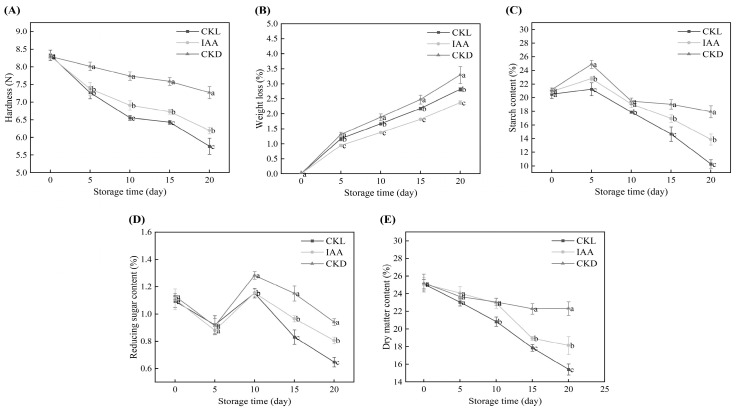
The figure illustrates the impact of IAA treatment on various parameters of potato tubers stored for 20 days, including weight loss (**A**), hardness (**B**), starch content (**C**), reducing sugar content (**D**), and dry matter content (**E**). Data are presented as mean ± standard error (*n* = 5). Different letters indicate significant difference in the treatment of each sampling time at *p* < 0.05.

The effect of IAA treatment on the firmness of whole potato tubers during storage is shown in Figure 5A. The firmness of potato tubers decreased with the storage time. The hardness of the CKL group decreased rapidly, dropping from the initial 8.33 N to 5.75 N during the storage period. After 20 days of storage, the hardness of the IAA group was significantly higher than that of the CKL group (*p* < 0.05). As shown in Figure 5B, compared with the CKL samples, the IAA-treated potato tubers exhibited a slower rate of weight loss during storage (*p* < 0.05). On day 20, the weight loss rates of the CKL and IAA groups reached 3.3% and 2.81%, respectively. Meanwhile, the starch content of potato tubers exhibited an initial increase followed by a subsequent decrease with the extension of storage time (Figure 5C). After 20 days of storage, the starch content of IAA-treated potato tubers was significantly higher than that of the CKL group (*p* < 0.05). As shown in Figure 5D, the content of reducing sugars in all samples showed a trend of initially decreasing, then increasing, and finally decreasing again. The content of reducing sugars in the IAA-treated samples was significantly higher than that in the CKL group on days 10–20 of storage (*p* < 0.05). Additionally, the dry matter content of all samples decreased with the extension of storage time (Figure 5E). After day 10, the dry matter content of the IAA group was significantly higher than that of the CKL group.

During the postharvest storage period, potato tubers typically undergo tissue softening and weight loss due to transpiration [43]. Previous studies have demonstrated that treatment with 1.0 mM IAA can maintain fruit firmness by downregulating genes associated with cell wall metabolism (*PG*, *PME31*, and two *PME63* isoforms) and ethylene-responsive transcription factors (*ERF3*, *ERF4*, and others), as well as alleviating weight loss in mango fruits [23]. Additionally, Chen et al. [25] demonstrated that exogenous IAA can preserve strawberry firmness by inhibiting genes associated with pectin depolymerization and cell wall component degradation. In this study, 1.5 mM IAA delayed the softening and weight loss of potato tubers during storage. The observed effect could be ascribed to exogenous IAA, which sustains potato firmness via the downregulation of cell wall metabolism-related genes and diminishes weight loss by suppressing ethylene transcription factors to attenuate metabolic activity. During the early storage period of potato tubers, the increase in starch content may be related to photosynthesis in the tuber epidermis. As reported by Qu et al. [44], when potato tubers are exposed to light, the chloroplasts in the epidermal cells carry out photosynthesis, converting light energy into chemical energy and synthesizing starch, which is stored in the form of starch granules. Interestingly, these starch granules can develop into chloroplasts with photosynthetic capabilities under light exposure. Consequently, after 10 days of storage, the starch content in the control group was lower than that in the IAA-treated group, whereas the Chl content was higher. This phenomenon may be attributed to the transformation of starch granules into chloroplasts in the control group, which led to the accumulation of Chl. Hendriks et al. [45] revealed that reducible sugars provide energy and carbon skeletons for Chl synthesis. In this study, it was found that IAA-treated potato tubers were able to maintain a high content of reducible sugars. This may be attributed to the slow metabolism of reducible sugars, which reduces the energy supply for the physiological and biochemical activities of potato tubers and consequently decreases Chl synthesis. As reported by Islam et al. [46], potato varieties with high dry matter content typically exhibit high starch content. This study further corroborated this observation, as the IAA-treated group demonstrated higher dry matter content and significantly higher starch content than the CKL group (Figure 5C,E). Intriguingly, the CKD group outperformed all other groups across every single metric. This finding was in line with the study by Gachango et al. [47], who demonstrated that potato tubers stored in the dark fare better than those stored under light conditions. This phenomenon could be attributed to the decreased rates of respiration and evaporation in dark storage.

Our results demonstrate that 1.5 mM IAA treatment effectively suppressed tuber greening and impaired postharvest quality deterioration in potatoes, as shown by higher firmness retention, lower weight loss, and maintained levels of starch and reducing sugars. The role of exogenous IAA in postharvest biology, however, is complex and highly context-dependent. While our observed senescence-delaying effect aligns with reports in mango [23] and strawberry [25], IAA application can promote softening in kiwifruit [37] and pear [38]. This duality underscores that the physiological outcome hinges on factors such as species, tissue type, and treatment protocol. In this study, the specific combination of potato tubers and the 1.5 mM IAA for 9 min treatment evidently induced a senescence-delaying program.

### 3.5. Analysis of the Transcriptome Profiles and DEGs of Potato Under IAA Treatment

In this study, the gene expression profiles of potato tubers treated with 1.5 mM IAA and the CKL after 20 days of storage were determined through transcriptome sequencing. Utilizing high-throughput sequencing, 37.55–62.87 million raw reads were obtained from six potato libraries. After the removal of low-quality reads, the results indicated that the clean reads accounted for 100% of the raw reads in the database, the mapped reads ranged from 85.65% to 91.62%, and the Q30 values ranged from 98.69% to 99.14% (Table S1). These values demonstrated that the filtered sequencing data were suitable for further analysis. The correlation coefficients between each pair of replicate samples revealed relatively consistent gene expression levels among the replicates (Figure S1).

**Figure 6:**
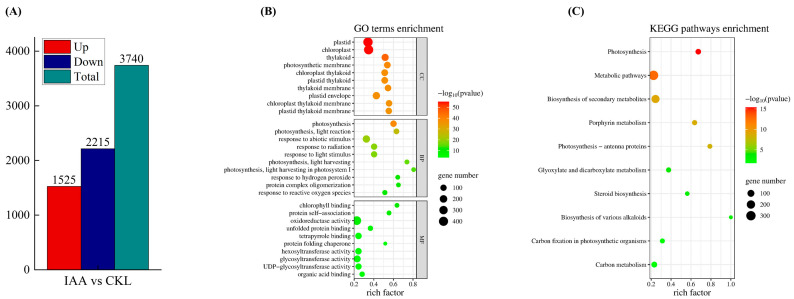
Number of up- and downregulation of DEGs in potato tubers treated with IAA (**A**). Gene ontology (GO) (**B**) and Kyoto encyclopedia of genes and genomes (KEGG) (**C**) enrichment analyses of these differential genes. In the GO and KEGG pathway enrichment analyses, the depth of the circle color indicates the degree of enrichment for each classification, while the size of the circle reflects the number of associated genes. KEGG pathways enrichment.

To identify the specific gene expression changes induced by IAA treatment, differentially expressed genes (DEGs) between the control and IAA-treated groups were compared after 20 days of storage. In this study, 3740 DEGs were identified, comprising 1525 upregulated and 2215 downregulated genes (Figure 6A).

To validate the effectiveness of transcriptomic analysis, the expression of six DEGs involved in the IAA-induced Chl synthesis pathway was analyzed via qRT-PCR (Figure S2). By comparing the expression levels of these DEGs between CKL and IAA-treated tubers at day 20 of storage, their expression patterns were found to be consistent with the transcriptomic data. Thus, the RNA-seq data possessed high reproducibility and reliability.

### 3.6. Gene Ontology (GO) Enrichment Analysis and KEGG Pathway Analysis of DEGs in Potato Tubers

In GO enrichment analysis, the DEGs were categorized into three groups: biological process (BP), molecular function (MF), and cellular component (CC). For each category, the top 10 subcategories with the smallest *p* values were selected (Figure 6B). Within the BP category, the subcategories related to tuber greening in potatoes included photosynthesis (107 DEGs), photosynthesis–light reaction (65 DEGs), response to abiotic stimulus (183 DEGs), response to radiation (100 DEGs), and response to light stimulus (96 DEGs). In the MF category, Chl binding (28 DEGs), tetrapyrrole binding (111 DEGs), and protein folding chaperone (15 DEGs) were linked to tuber greening. For the CC category, chloroplast (415 DEGs) and photosynthetic membrane (127 DEGs) were significantly enriched and related to greening.

KEGG pathway analysis revealed that 16 pathways were significantly enriched. DEGs associated with the potato greening process were primarily involved in porphyrin metabolism, photosynthesis–antenna proteins, biosynthesis of various alkaloids, and carbon fixation in photosynthetic organisms (Figure 6C).

### 3.7. Chl Biosynthesis Pathway in Potato Greening Process

**Figure 7:**
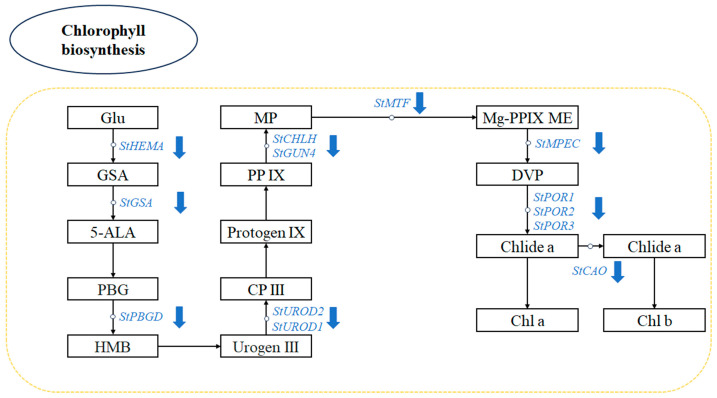
Identification of differentially expressed genes (DEGs) associated with chlorophyll biosynthesis as determined by a KEGG pathway analysis in CKL 20 d, and IAA 20 d sample groups. Blue arrow indicates downregulation of DEGs in the CKL 20 d vs. IAA 20 d treated group comparison.

Chl synthesis in plant cells is a complex biochemical process with intricate regulation, involving a series of enzyme-catalyzed reactions (Figure 7). The rate-limiting step of this pathway is catalyzed by glutamyl-tRNA reductase (GluTR), encoded by the *HEMA* gene, converting glutamyl-tRNA to glutamate-1-semialdehyde (GSA) [48]. Glutamate-1-semialdehyde aminotransferase, encoded by the *GSA* gene, generates 5-aminolevulinic acid, a critical branching point for Chl and heme metabolism [49]. Porphobilinogen deaminase (PBGD) regulates metabolic flux through porphyrinogen polymerization, whereas the magnesium chelatase catalytic subunit (*CHLH*) and GUN4 protein facilitate magnesium ion insertion into protoporphyrin IX and its cellular transport, respectively [50,51]. Light-dependent protochlorophyllide oxidoreductase (POR) controls the greening of etiolated tissues via light signaling [52]. In IAA-treated potato tubers, the expression levels of *StPBGD*, *StHEMA*, and *StPOR* are reduced. Moreover, the downregulation of genes involved in magnesium ion insertion during Chl synthesis, such as *StCHLH* and *StGUN4*, results in the inhibition of Chl synthesis (Table 1).

### 3.8. Exogenous IAA Suppresses Chl Biosynthesis Through a Coordinated Multi-Hormonal Regulatory Network

**Figure 8:**
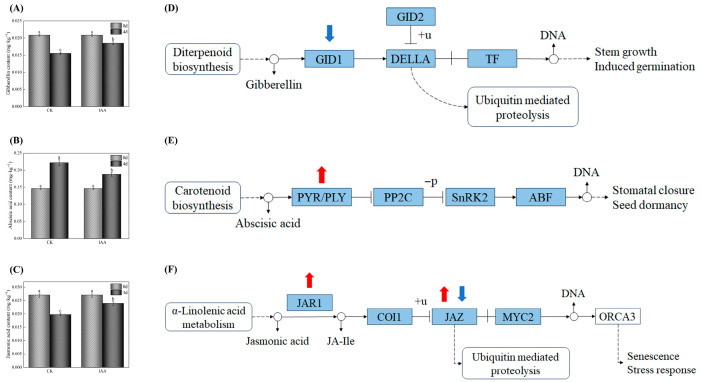
Endogenous phytohormone levels and transcriptomic regulation of their signaling pathways in potato tubers. Contents of gibberellin (**A**), abscisic acid (**B**), and jasmonic acid (**C**) quantified in CKL and IAA-treated tubers at 0 and 20 days of storage. Identification of differentially expressed genes (DEGs) associated with plant hormone signal transduction as determined by a KEGG pathway analysis in CKL 20 d, and IAA 20 d sample groups. Gibberellin signaling pathway (**D**). Jasmonate signaling pathway (**E**). Abscisic acid signaling pathway (**F**). Red arrow indicates upregulation of DEGs and blue arrow indicates downregulation of DEGs in the CKL 20 d vs. IAA 20 d treated group comparison. CKL represents the light control group. Data are presented as mean ± standard error (*n* = 3). Different letters indicate significant difference in the treatment of each sampling time at *p* < 0.05.

Chl metabolism in plants is under intricate regulation by various endogenous and exogenous factors [53]. This study systematically elucidates the multi-pathway mechanism by which exogenous IAA suppresses greening in potato tubers by modulating the endogenous hormone network (Figure 8). Regarding the GA pathway, light exposure suppressed GA biosynthesis in tubers, whereas IAA treatment increased GA levels (Figure 8A). Previous studies have indicated that a decrease in endogenous GA content promotes Chl accumulation [6], likely through the accumulation of DELLA proteins, which repress PIF transcriptional activity and thereby relieve the inhibition of Chl biosynthesis-related genes [54]. In the ABA pathway, ABA content in tubers gradually increased during storage, while IAA treatment significantly reduced ABA levels (Figure 8B). Although ABA has been shown to enhance Chl synthesis in duckweed [55], this study observed upregulation of *PYR/PYL* gene expression (Table 1), suggesting that activated *ABF* transcription factors may bind to *ABRE* elements in the promoters of Chl synthesis genes and inhibit their expression [56]. By reducing both ABA content and its signaling, IAA treatment effectively delayed tuber greening. In the JA pathway, IAA treatment markedly increased JA content (Figure 8C) and altered the expression of JA signaling-related genes (Table 1). *JA-Ile*, the bioactive form of JA, acts through the COI1–JAZ–MYC2 signaling module to activate defense-related genes and suppress photosynthesis [57,58,59]. Consistent with the report by Xue et al. (2025) that reduced JA levels enhance Chl accumulation [60], this study also confirmed a negative correlation between JA and Chl content. Therefore, exogenous IAA coordinately regulates the levels and signaling of GA, ABA, and JA pathways, forming an interconnected regulatory network that collectively inhibits greening in potato tubers during storage.

## 4. Conclusions

This study investigated the inhibitory effects of exogenous IAA on the greening of postharvest potato tubers and its underlying molecular mechanisms through physiological and transcriptomic analyses. Results demonstrated that a 9 min treatment with 1.5 mM IAA significantly reduced the Chl content, maintained elevated a* values, and effectively inhibited greening in potato tubers. Additionally, IAA treatment notably delayed the softening and weight loss of potatoes while preserving elevated levels of starch and reducing sugars. Transcriptomic analysis further elucidated the molecular regulatory mechanisms of IAA treatment, revealing that IAA significantly downregulated the expression of key Chl biosynthesis genes (e.g., *HEMA1*, *CHLH*, and *GUN4*) and activated critical genes in the GA, JA, and ABA signaling pathways (e.g., *StGID1*, *StJAR1*, *StPYR1* and *StPYL4*), thereby inhibiting Chl synthesis. These results indicate that exogenous IAA treatment can inhibit greening while maintaining the nutritional value of potatoes, offering a novel approach for light-sensitive agricultural products like potatoes. However, it is crucial to note that IAA is a plant growth regulator whose postharvest application to fresh edible produce is not widely approved. Before any commercial adoption can be considered, further research is paramount to evaluate IAA residue levels in tubers for food safety, address regulatory requirements, and gauge consumer acceptance. Future research should focus on monitoring IAA residue levels in treated tubers to ensure food safety, evaluating the economic feasibility of integrating this treatment into existing postharvest handling systems, and validating its efficacy across various potato cultivars and commercial storage environments.

## Figures and Tables

**Table 1:** Differentially expressed genes in potato tubers greening process under IAA treatment.

Gene ID	Gene Name	Log2(FC)	Description
PGSC0003DMG400006381	*StHEMA*	−4.3692	Glutamyl-tRNA reductase
PGSC0003DMG400032546	*StGSA*	−2.183	Glutamate-1-semialdehyde 2,1-aminomutase, chloroplastic
PGSC0003DMG400022126	*StPBGD*	−1.9418	Porphobilinogen deaminase
PGSC0003DMG400027602	*StUROD1*	−2.755	Uroporphyrinogen decarboxylase, chloroplastic
PGSC0003DMG402021263	*StUROD2*	−2.1133	Uroporphyrinogen decarboxylase
PGSC0003DMG400027276	*StCHLH*	−7.8366	Mg protoporphyrin IX chelatase
PGSC0003DMG400014243	*StMTF*	−3.7492	S-adenosyl-L-methionine Mg-protoporphyrin IX methyltranserase
PGSC0003DMG400007188	*StMPEC*	−6.8581	Desaturase
PGSC0003DMG400015356	*StPOR1*	−6.6087	NADPH:protochlorophyllide oxidoreductase
PGSC0003DMG400025007	*StPOR2*	−5.6145	NADPH:protochlorophyllide oxidoreductase
PGSC0003DMG400018351	*StPOR3*	−3.0866	NADPH:protochlorophyllide oxidoreductase
PGSC0003DMG400017570	*StCAO*	−2.8109	Chlorophyll synthase
PGSC0003DMG400027013	*StGUN4*	−5.3941	Tetrapyrrole-binding protein, chloroplast
PGSC0003DMG400034947	*StHMGR*	−5.5889	HMGR CoA reductase
PGSC0003DMG400003849	*StGID1*	−1.2406	GID1-like gibberellin receptor
PGSC0003DMG400033879	*StJAR1*	1.484	Jasmonic acid-amino acid-conjugating enzyme
PGSC0003DMG400015667	*StPtoR1*	−2.7824	Pto-responsive gene 1 protein
PGSC0003DMG400004367	*StSRP1*	4.9798	Salt responsive protein 1
PGSC0003DMG400032119	*StJAZ3*	1.506	Jasmonate ZIM-domain protein 3
PGSC0003DMG400022888	*StSRP1*	−1.2682	Salt responsive protein 1
PGSC0003DMG400002100	*StPYR1*	1.0557	Abscisic acid receptor PYR1
PGSC0003DMG400029194	*StPYL4*	2.8127	Abscisic acid receptor PYL4

## Data Availability

The original contributions presented in the study are included in the article, further inquiries can be directed to the corresponding author.

## Supplementary Materials

The following supporting information can be downloaded at: https://www.mdpi.com/article/10.3390/foods14234031/s1, Figure S1: Principal component analysis (PCA) reveals transcriptome variations in potato tubers under different treatment conditions, Figure S2: RT-qPCR verification results of some DEGs, Table S1: Primer sequences used for RT-qPCR analysis, Table S2: Transcriptome sequencing quality statistics of potato tubers under different treatment conditions.
