# Supplementary material for: Physiological and Transcriptomic Analyses of IAA-Induced Inhibition of Chlorophyll Formation in Potato Tubers Post-Harvest
Source: Foods. 2025 Nov 25;14(23):4031. doi: 10.3390/foods14234031 (PMC12691855; doi:10.3390/foods14234031)
Supplement: Supplementary file 1 [file foods-14-04031-s001.zip › Supplementary Figures.pdf]

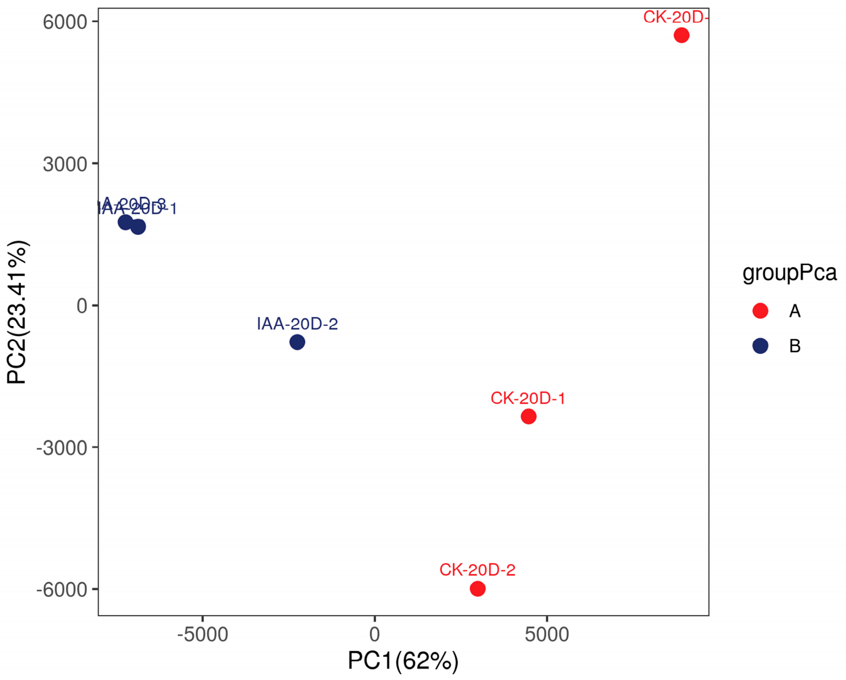

Figure S1. Principal component analysis (PCA) reveals transcriptome variations in potato tubers under different treatment conditions

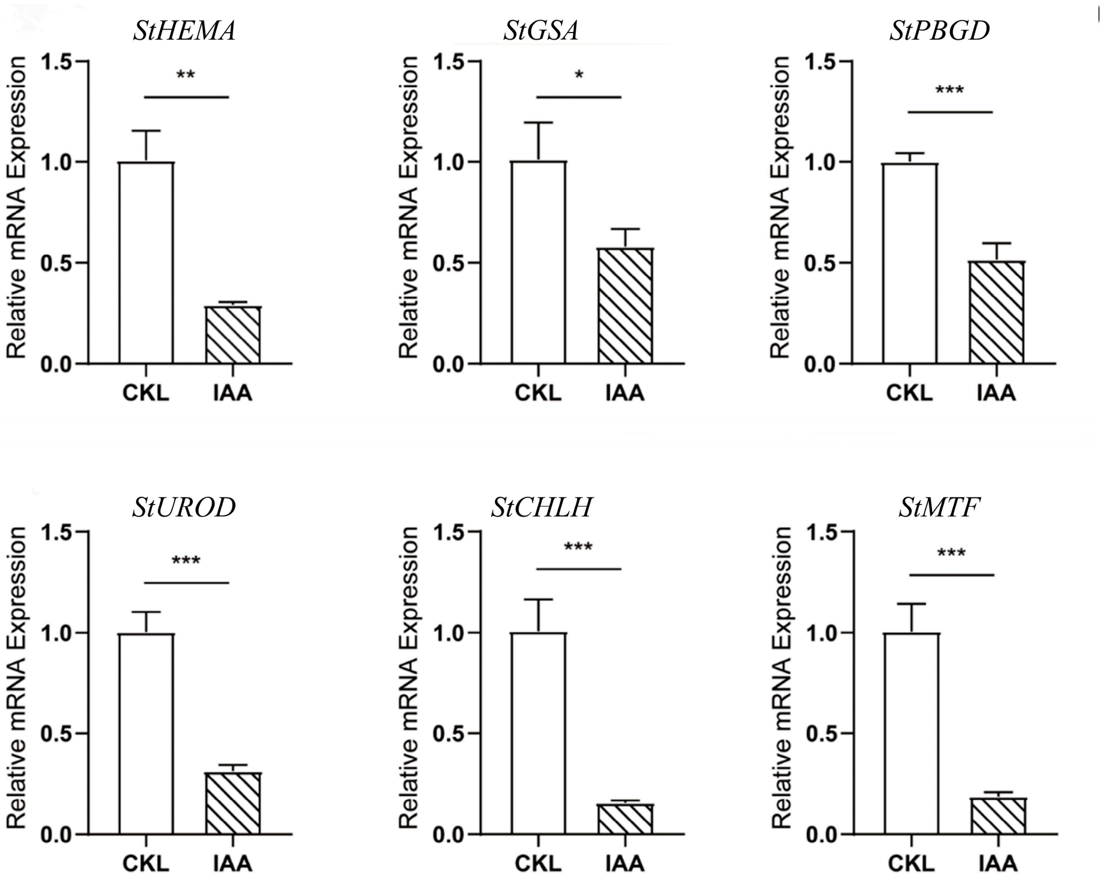

Figure S2. RT-qPCR verification results of some DEGs.
