# Supplementary material for: Physiological and Transcriptomic Analyses of IAA-Induced Inhibition of Chlorophyll Formation in Potato Tubers Post-Harvest
Source: Foods. 2025 Nov 25;14(23):4031. doi: 10.3390/foods14234031 (PMC12691855; doi:10.3390/foods14234031)
Supplement: Supplementary file 1 [file foods-14-04031-s001.zip › Supplementary Tables.pdf]

| **Gene name** | **Forward Primer** | **Reverse Primer** |
|---|---|---|
| *StHEMA* | ATGGCTATTCCAACAGCATTT | TTATTGTTTTTGAGTTTGCC |
| *StGSA* | ATGGCTGCAGTTAAATGGTGT | TTAAATTTGCTTCAAAACTTT |
| *StPBDG* | ATGGAGAAATTGTCTACTCTAA | TTAGTTGTTGTTGTTGTTGTTG |
| *StUROD* | ATGTCTTGTATTTACAGCTC | CTAATACCTAGAACCTTTG |
| *StMTF* | ATGGCTTTCTCCTCGCCGCT | CTAAGCAGGGACAGCTTCAATAG |
| *StCHLH* | ATGGCTTCTTTGGTTTCTT | TTATCGATCGATTCC |

**Table S1** Primer sequences used for RT-qPCR analysis

| Sample name | Raw_reads | Clean_reads | Clean_bases | Total mapped | Total mapped(%) | Error(%) | Q20(%) | Q30(%) | GC(%) |
|---|---|---|---|---|---|---|---|---|---|
| CK 20D_1 | 37551394 | 37551394 | 5.62G | 34205961 | 91.09% | 0.02 | 99.67 | 98.77 | 43.99 |
| CK 20D_2 | 46243156 | 46243156 | 6.88G | 42006815 | 90.84% | 0.02 | 99.64 | 98.69 | 45.53 |
| CK 20D_3 | 62865068 | 62865068 | 9.23G | 53844118 | 85.65% | 0.02 | 99.77 | 99.14 | 39.67 |
| IAA 20D_1 | 50009212 | 50009212 | 7.48G | 44826613 | 89.64% | 0.02 | 99.68 | 98.77 | 44.06 |
| IAA 20D_2 | 56147416 | 56147416 | 8.4G | 51441361 | 91.62% | 0.02 | 99.69 | 98.8 | 45.35 |
| IAA 20D_3 | 53195956 | 53195956 | 7.94G | 47956776 | 90.15% | 0.02 | 99.7 | 98.88 | 44.81 |

**Table S2** Transcriptome sequencing quality statistics of potato tubers under different treatment conditions
